# Effects of Bioinsecticidal Aegerolysin-Based Cytolytic Complexes on Non-Target Organisms

**DOI:** 10.3390/toxins13070457

**Published:** 2021-06-30

**Authors:** Anastasija Panevska, Gordana Glavan, Anita Jemec Kokalj, Veronika Kukuljan, Tomaž Trobec, Monika Cecilija Žužek, Milka Vrecl, Damjana Drobne, Robert Frangež, Kristina Sepčić

**Affiliations:** 1 Department of Biology, Biotechnical Faculty, University of Ljubljana, 1000 Ljubljana, Slovenia; anastasija.panevska@bf.uni-lj.si (A.P.); gordana.glavan@bf.uni-lj.si (G.G.); anita.jemec@bf.uni-lj.si (A.J.K.); damjana.drobne@bf.uni-lj.si (D.D.); 2 Department of Biotechnology, University of Rijeka, 51000 Rijeka, Croatia; veronika.kukuljan@gmail.com; 3 Institute of Preclinical Sciences, Veterinary Faculty, University of Ljubljana, 1000 Ljubljana, Slovenia; tomaz.trobec@vf.uni-lj.si (T.T.); monika.zuzek@vf.uni-lj.si (M.C.Ž.); milka.VreclFazarinc@vf.uni-lj.si (M.V.)

**Keywords:** adverse effects, aegerolysins, *Apis mellifera*, bioinsecticides, erylysin A, MACPF protein, non-target organisms, oyster mushroom, *Porcellio scaber*, toxicity

## Abstract

Aegerolysin proteins ostreolysin A6 (OlyA6), pleurotolysin A2 (PlyA2) and erylysin A (EryA) produced by the mushroom genus *Pleurotus* bind strongly to an invertebrate-specific membrane sphingolipid, and together with a protein partner pleurotolysin B (PlyB), form transmembrane pore complexes. This pore formation is the basis for the selective insecticidal activity of aegerolysin/PlyB complexes against two economically important coleopteran pests: the Colorado potato beetle and the western corn rootworm. In this study, we evaluated the toxicities of these aegerolysin/PlyB complexes using feeding tests with two ecologically important non-target arthropod species: the woodlouse and the honey bee. The mammalian toxicity of the EryA/PlyB complex was also evaluated after intravenous administration to mice. None of the aegerolysin/PlyB complexes were toxic against woodlice, but OlyA6/PlyB and PlyA2/PlyB were toxic to honeybees, with 48 h mean lethal concentrations (LC_50_) of 0.22 and 0.39 mg/mL, respectively, in their food. EryA/PlyB was also tested intravenously in mice up to 3 mg/kg body mass, without showing toxicity. With no toxicity seen for EryA/PlyB for environmentally beneficial arthropods and mammals at the tested concentrations, these EryA/PlyB complexes are of particular interest for development of new bioinsecticides for control of selected coleopteran pests.

## 1. Introduction

Mushrooms from the genus *Pleurotus* (oyster mushrooms) have recently been shown to produce proteins that can act as potent and selective bioinsecticides against two economically very important coleopteran pests: the western corn rootworm (*Diabrotica v. virgifera*; WCR) and the Colorado potato beetle (*Leptinotarsa decemlineata*; CPB) [1]. These insecticidal effects are mediated by a complex formed by two proteins (i) one that belongs to the family of aegerolysins that share high similarities in their amino-acid sequences (78–97%); namely, ostreolysin A6 (OlyA6), pleurotolysin A2 (PlyA2) or erylysin A (EryA) [2]; and (ii) one that has a membrane-attack complex/perforin (MACPF) domain; namely, pleurotolysin B (PlyB) [3,4]. 

The biological activities of the OlyA6/PlyB, PlyA2/PlyB and EryA/PlyB complexes result from their specific recognition of and binding to cell membrane sphingolipids. The subtle differences in the primary structures of these highly similar *Pleurotus* aegerolysins dictate their affinities and selectivities towards these sphingolipids. For example, OlyA6, PlyA2 and PlyA recognize sphingomyelin/cholesterol complexes [4,5,6], whereas EryA lacks this activity [1,7]. On the other hand, all *Pleurotus* aegerolysins that have been tested, including EryA, can recognize and strongly bind to artificial lipid vesicles that contain equimolar amounts of cholesterol and ceramide phosphoethanolamine (CPE) [1,7]. This is the major sphingolipid in invertebrate cell membranes, while it is absent in other taxa [8]. For OlyA6 and PlyA2, this interaction is even 1000-fold stronger than the interaction with sphingomyelin-containing membranes [7,9].

It has been shown that the binding of OlyA6 or PlyA to membrane sphingomyelin/cholesterol domains recruits the MACPF-partnering protein PlyB [4,5]. This association of PlyB with the membrane-bound aegerolysin results in major conformational changes that promote the penetration of the lipid bilayer and the formation of 13-meric bi-component transmembrane pores. Similarly, OlyA6, PlyA2 and EryA can bind to and permeabilize (when combined with PlyB) artificial lipid vesicles and biological membranes containing physiologically relevant concentrations (1–5 mol%) of CPE [1,10]. Indeed, the insecticidal activities of these OlyA6/PlyB, PlyA2/PlyB and EryA/PlyB complexes towards WCR and CPB appear to be due to the formation of transmembrane pores in the insect midgut epithelium [1], similar to the pores that have been described in sphingomyelin/cholesterol membrane systems [4,5].

Due to their specific interactions with their membrane lipid receptor CPE, these aegerolysin-based bicomponent proteinaceous complexes might represent alternatives to currently used bioinsecticides, such as the proteinaceous crystal toxins (i.e., Cry toxins) from the bacterium *Bacillus thuringiensis*. Transgenic potato cultivars and corn hybrids that express Cry toxins (e.g., Bt potatoes, Bt maize) have been developed to provide resistance to CPB and WCR, and have been used in commercial cultivation in the USA since 1995 and 2003, respectively [11,12,13,14,15]. One of the Cry toxins used against WCR is the binary Cry34Ab1/Cry35Ab1 complex, where, structurally, Cry34Ab1 belongs to the aegerolysin protein family [16,17]. The insecticidal effects of the Cry toxins are due to their binding to a variety of protein receptors in the insect midgut epithelium [18]. However, the insects can develop resistance to Bt maize through different mechanisms, where reduced Cry toxin binding as a result of mutation of the protein receptor gene is the major mechanism of resistance in field-evolved resistance to Cry toxins [19,20]. As a consequence, the current management of WCR using these Cry toxins is challenged by the development of resistance to all of the currently available Bt maize [21].

Interestingly, the OlyA6/PlyB, PlyA2/PlyB and EryA/PlyB protein complexes are inactive against other insect pests, including: the mealworm beetle, *Tenebrio molitor* (Coleoptera); the spotted wing fruit fly, *Drosophila suzukii* (Diptera); the greater wax moth, *Galleria melonella* (Lepidoptera); and the grain aphid, *Sitobion avenae* (Hemiptera) [1]. This specificity against WCR and CPB appears to be due to the characteristic acidic environment in the midgut of WCR [22] and CPB [23], which is optimal for OlyA6 binding to membrane lipids [24]. In addition to effects on WCR and CPB, it has been shown previously that a native OlyA6/PlyB isolate can induce cardiorespiratory arrest in mice after intravenous administration, and that these toxic effects are most likely to be the result of formation of OlyA6/PlyB pores in membranes of erythrocytes and other mammalian cells [25].

To further evaluate the potential toxicities of these *Pleurotus* aegerolysin-based bicomponent protein complexes, we investigated the effects of OlyA6/PlyB, PlyA2/PlyB and EryA/PlyB on the feeding and survival rates of two non-target arthropod species that perform important ecological functions: woodlice (*Porcellio scaber*) and honeybees (*Apis mellifera carnica*). By non-target species we describe the non-intended victim of an application of a bioinsecticide. Woodlice are an established model organism for terrestrial ecotoxicity testing because they have an important ecosystem function in the decomposition of organic material [26]. Honeybees are ecologically and economically very important as crop pollinators and honey producers, which are now threatened by disease, loss of food sources and environmental contamination with pesticides [27]. EryA is the only *Pleurotus*-derived aegerolysin candidate that shows exclusive specificity for the insect-specific lipid receptor CPE [1,7], and that does not interact with sphingomyelin, which is the dominant sphingolipid in mammalian cell membranes. We therefore also evaluated the toxicity of the EryA/PlyB complex upon intravenous administration to mice.

## 2. Results

### 2.1. Toxicity Tests with Woodlice (Porcellio scaber)

Healthy adult woodlice were exposed to the test substances over 7 days of feeding on potato (*Solanum tuberosum*) leaves. Based on our previous experiments with *Pleurotus* aegerolysin/PlyB complexes with WCR and CPB [1], cut fresh leaves were immersed for 5 min in solutions of the aegerolysins (0.5 mg/mL; OlyA6, PlyA2, EryA) with PlyB (0.04 mg/mL), to provide 9.0 µg/cm^2^ aegerolysin/PlyB on each leaf surface.

**Figure 1:**
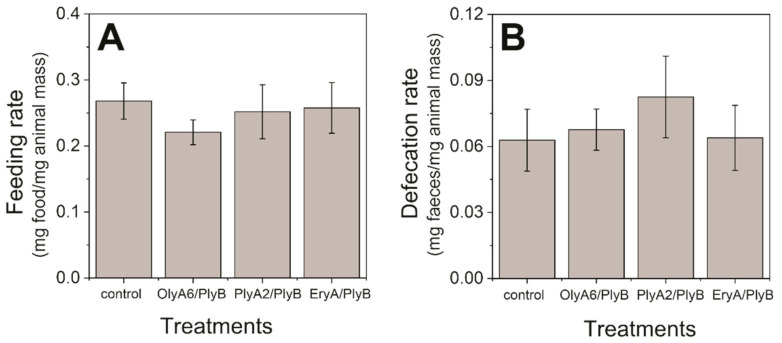
Feeding (**A**) and defecation (**B**) rates of the woodlice exposed to 9.0 µg/cm^2^ aegerolysin/pleurotolysin B (PlyB) complex (0.5 mg ostreolysin A6 (OlyA6), pleurotolysin A2 (PlyA2) or erylysin A (EryA) supplemented with 0.04 mg/mL PlyB) for 7 days. Data are means ± standard error (*n* = 10 woodlice exposed per treatment).

There were no statistically significant differences between the feeding rates and defecation rates of the control woodlice and those exposed to these treatments (Figure 1; *p* ˃ 0.05; Mann–Whitney tests). There was also no mortality of these woodlice for any of these treatments (i.e., OlyA6/PlyB, PlyA2/PlyB, EryA/PlyB). We can thus conclude that none of the treatments affected the feeding physiology and survival of these woodlice.

### 2.2. Toxicity Tests with Honey Bees (Apis mellifera carnica)

Adult worker honeybee foragers were exposed to the test substances over 48 h of feeding on 1.5 M sucrose in dechlorinated water, with the treatment concentrations initially chosen again based on our previous experiments with *Pleurotus* aegerolysin/PlyB complexes on WCR and CPB [1]. The sucrose solutions were offered to the honeybees ad libitum as the control (no addition), the single aegerolysins, or OlyA6/PlyB, PlyA2/PlyB or EryA/PlyB, with dechlorinated water with no additions also provided. These were all renewed with freshly prepared solutions after 24 h.

#### 2.2.1. Toxicities of Aegerolysin and Their Mixtures with PlyB

**Figure 2:**
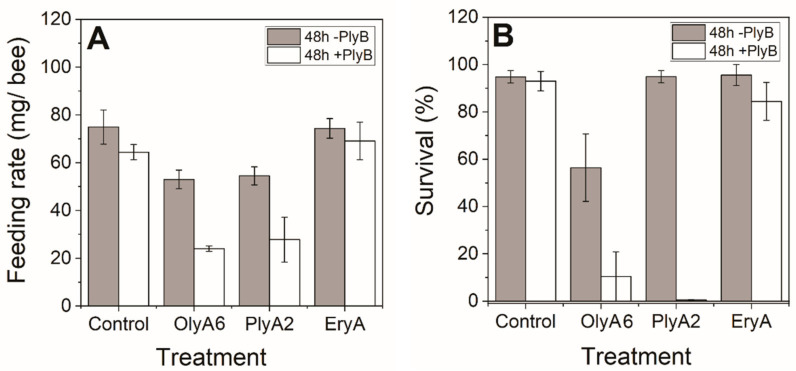
Feeding (**A**) and survival (**B**) rates of honeybees feeding on 0.5 mg/mL OlyA6, PlyA2 or EryA without (grey bars) and with (white bars) 0.04 mg/mL PlyB over 48 h. Data are means ± standard error (*n* = 3 groups of bees per treatment).

The feeding rates of the honeybees were evaluated as an average feeding rate/honeybee over the 48 h of the experiments, initially for exposure to the control (no additions) and to 0.5 mg/mL aegerolysins OlyA6, PlyA2 and EryA, each without and with 0.04 mg/mL PlyB. These were lower for honeybees fed with the aegerolysin/PlyB complexes compared to those fed with both the control and the aegerolysins without PlyB (Figure 2A). The greatest decrease in 48 h food consumption compared to the relevant aegerolysin alone was seen for OlyA6/PlyB and PlyA2/PlyB. For the aegerolysins alone, the lowest feeding rates were seen for OlyA6 and PlyA2.

Similarly, lower survival rates were seen for honeybees after this feeding exposure to OlyA6/PlyB and PlyA2/PlyB (11%, 0%, respectively) compared to both the control and the relevant aegerolysin alone (i.e., without PlyB) (Figure 2B). For EryA/PlyB at 48 h, there remained relatively high survival (85%), particularly considering that according to the OECD TG 245 protocol, 15% mortality is allowed in the controls. However, the survivals of the honeybees treated with 0.5 mg/mL OlyA6, PlyA2 and EryA without PlyB were 57%, 95% and 96%, respectively.

#### 2.2.2. Dose–Response Toxicity Testing of OlyA6/PlyB and PlyA2/PlyB

As the aegerolysin complexes showed reduced feeding rates and survivals (except EryA/PlyB) (Figure 2), we performed dose–response analysis to calculate the concentrations for 50% (lethal) effects (LC_50_) for OlyA6/PlyB and PlyA2/PlyB.

**Figure 3:**
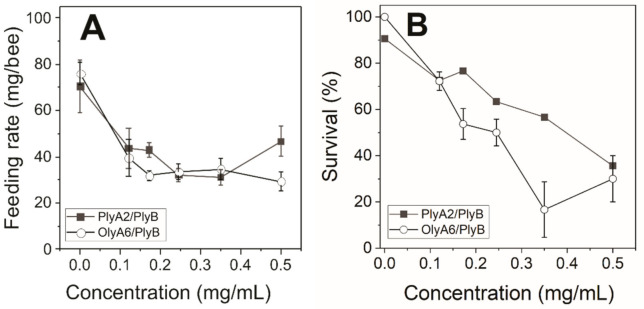
Feeding (**A**) and survival (**B**) rates of honeybees feeding on increasing concentrations of OlyA6/PlyB and PlyA2/PlyB for 48 h. The sigmoid curves for the data fitting for the LC_50_ values are shown in Figure S1. Data are means ± standard error (*n* = 3 groups of bees exposed per treatment).

The feeding rates of the honeybees with the addition of OlyA6/PlyB and PlyA2/PlyB were around half of those for the controls for all of the concentrations tested (Figure 3A). In contrast, the survival of these honeybees decreased with increasing concentrations of OlyA6/PlyB and PlyA2/PlyB (Figure 3B). The LC_50_ values calculated by fitting of these data to a sigmoid function (Figure S1) were 0.22 mg/mL and 0.39 mg/mL for OlyA6/PlyB and PlyA2/PlyB, respectively.

### 2.3. Determination of Acute Toxicity of EryA/PlyB in Mice

While the OlyA6/PlyB, PlyA2/PlyB and EryA/PlyB protein complexes are active against WCR and CPB, they have instead been shown to be inactive against a number of insect pests [1]. The present study also defines no activity against woodlice, but toxicity towards bees, except for EryA/PlyB. Indeed, EryA is the only *Pleurotus*-derived aegerolysin that shows exclusive specificity for the insect-specific lipid receptor CPE [1,7]. We previously showed that intravenous OlyA6/PlyB can induce cardiorespiratory arrest in mice [25], and therefore we next examined potential toxicity of the EryA/PlyB upon intravenous administration to mice.

None of the EryA/PlyB doses tested (0.5, 1.0, 3.0 mg/kg) caused visible signs of systemic intoxication in mice. Indeed, all of the animals survived with no changes in behavior, locomotion and food and water intake over 24 h after intravenous administration.

#### 2.3.1. Body and Organ Mass

The body mass of these mice and the relative mass of the main organs are summarized in Table 1. These acute (24 h) treatments with EryA/PlyB also had no effects on body mass or relative organ mass.

#### 2.3.2. Histological Evaluation

**Figure 4:**
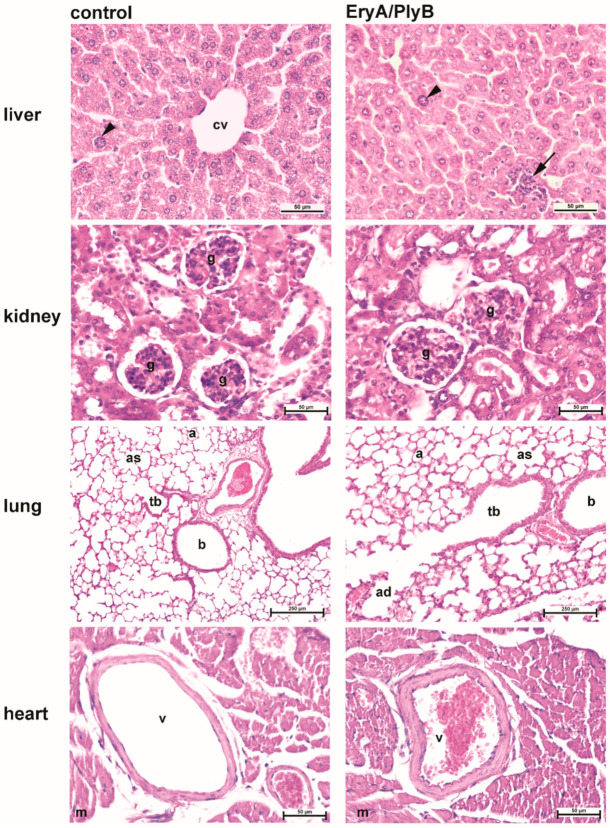
Representative photomicrographs of hematoxylin/eosin-stained liver, kidney, lung and heart sections 24 h after control and 3 mg/kg EryA/PlyB intravenous injection of the mice. Liver: arrowheads, karyomegaly (polyploidization); arrow, inflammatory cell infiltration; cv, central vein. Kidney: g, glomerulus. Lungs: a, alveoli; ad, alveolar duct; ac, alveolar sac; b, bronchiole; tb, terminal bronchiole. Heart: m, myocardium; v, coronary vessel. Scale bars, 50 µm (liver, kidney, heart); 250 µm (lung).

Representative histological sections of the liver, renal cortex, lung and left ventricle (with coronary vessel) from these mice under the control and 3 mg/kg EryA/PlyB intravenous treatments are shown in Figure 4. There were no obvious histological differences seen between these treatments.

In the liver, there was inflammatory cell infiltration with the EryA/PlyB-treated mice, although these cells were also seen in the individual liver sections of the control mice (data not shown). Furthermore, the observed karyomegaly (polyploidization) seen in the liver sections can be described as an aging change [28], and there was no apparent treatment-associated increase in this liver polyploidization (Figure 4).

For the sections from the cortex of the kidney, the lungs and the left ventricle with coronary vessels of the heart, EryA/PlyB-treated mice showed no obvious signs of acute toxicity (e.g., endothelial damage with severe perivascular oedema and hemorrhages) as reported with other aegerolysin-based binary pore-forming cytolytic protein complexes [25,29]. However, some minor differences suggestive of an endothelial response were noted in EryA/PlyB-treated mice, including a slight deviation in glomerular cell density (cf. kidney photomicrographs) and minor thickening of interalveolar septa (cf. lung photomicrographs) and myocardial vessel wall (cf. cardiac photomicrographs). Yet, these changes were not accompanied by inflammatory infiltrates/necrosis (Figure 4).

## 3. Discussion

In view of their specific interactions with insect-specific membrane lipid receptors, the aegerolysin-based cytolytic complexes produced by the fungal genus *Pleurotus* might represent new and prospective candidates against WCR and CPB in the further development of biopesticides produced by genetically modified corn and potato plants. However, to further evaluate the environmental toxicities of any new (bio)insecticide, it is very important to determine its toxicity towards non-target organisms that are likely to be directly or indirectly exposed to the particular toxin. Indeed, this is a particular consideration in terms of organisms that have beneficial environmental functions (e.g., decomposers and pollinators) or that are natural enemies of agricultural pests [30]. As an example, protein-binding aegerolysin-based Cry34/Cry35Ab1 complexes from *B. thuringiensis* have been tested on a wide range of non-target animals, including various insects, a nematode, a crustacean, a fish, a bird and mammal (rodent) species. This complex showed no toxic or lethal effects on any of the animals tested at the concentrations predicted to be present in the environment [31,32,33,34].

The present toxicity study on the selected non-target species showed no effects of any of these aegerolysin/PlyB complexes against the woodlice. This was seen despite the slightly acidic gut pH of this terrestrial isopod [35], which would be favorable for aegerolysin binding [24]. BLAST searches against databases for the Isopods using the *Drosophila melanogaster* CPE synthase gene and protein sequences did not reveal the presence of any homologue in woodlice [36]. The absence of a CPE synthase might be the reason for the absence of CPE and of any toxic effects of the aegerolysin/PlyB complexes in woodlice.

On the other hand, OlyA6/PlyB and PlyA2/PlyB were toxic against these adult forager honeybees, with 48 h LC_50_ values of 0.22 mg/mL and 0.39 mg/mL, respectively, when applied in their food. Although it is not easy to directly compare these data with those obtained with WCR larvae due to the different modes of exposure, OlyA6/PlyB and PlyA2/PlyB appear to be less toxic for the bees. Indeed, the 3-day LD_50_ values for WCR larvae for OlyA6/PlyB and PlyA/PlyB have been estimated to be 0.058 mg/mL and 0.055 mg/mL, respectively [1]. These are lower in the WCR larvae compared to the bees, indicating their likely greater effectiveness against WCR. On the other hand, this might simply reflect the use of adult bees in the present study, and not larvae, that should be tested for their susceptibility on aegerolysin-based bioinsecticidal complexes in the future. Indeed, our previous studies showed that WCR adults were also more resistant to aegerolysin/PlyB insecticidal protein mixtures than the WCR larvae [1]. Honeybees have been reported to have CPE in their cell membranes [37] and the gut pH of the adult forager bees is acidic [38]. This would thus favor interactions between the aegerolysin-based cytolytic complexes and the gut membranes, with the potential for the consequent toxic effects. However, unlike OlyA6/PlyB and PlyA2/PlyB, the EryA/PlyB complexes that are effective against WCR larvae and adults, and also CPB larvae [1], were not toxic to the bees at the concentrations tested here. This might be expected to be due to the lower interactions of EryA with membrane-bound CPE, and consequently a lower lytic activity, as has been reported using artificial and biological lipid systems [1]. The decreased survival of honeybees exposed to 0.5 mg/mL OlyA6 alone, observed in this study, can be due to the perturbation of the honeybee midgut apical membranes structure induced by the OlyA6 alone [4,39,40], especially considering that the tested proteins were applied at very high doses. The same doses of single aegerolysins, however, did not have deleterious effects on CPB and WCR in toxicity assays [1].

Furthermore, in mice, these EryA/PlyB complexes were not toxic and did not induce visible histological alterations even when applied intravenously at the highest dose tested (3 mg/kg body mass EryA/PlyB). This is in agreement with the lack of EryA binding to the main mammalian membrane sphingolipid, sphingomyelin [1,7]. In contrast, the native OlyA6/PlyB isolate (also known as ostreolysin) that binds to membrane sphingomyelin/cholesterol domains [4,39] was lethal to mice after intravenous administration, with a 24-h LD_50_ of 1170 μg/kg [25]. In rats, OlyA6/PlyB induces a transient rise in arterial blood pressure, followed by a progressive fall in blood pressure, which is associated with noticeable bradycardia and myocardial ischemia. The drop in blood pressure was accompanied by ventricular extrasystoles, which are associated with marked hyperkalemia. Moreover, in both rat and pig, sub-micromolar concentrations of OlyA6/PlyB induced a concentration-dependent increase in aortic ring tension [29,41]. It is worth noting that although OlyA6/PlyB complexes are lethal to mice after intravenous administration, as these proteins are derived from an edible mushroom, the more standard oral intake would lead to their degradation in the digestive tract.

## 4. Conclusions

In conclusion, although the affinity of EryA for CPE is lower than that of OlyA6 and PlyA2, EryA is the only one of these that shows exclusive specificity for the insect-specific lipid receptor, CPE. Added to this, there is the absence of interactions of EryA with mammalian sphingolipids [1,7] and the lack of toxicity for rodents and beneficial insects (e.g., the honeybees in the present study). Therefore, complexes with EryA are of particular interest for further development and improvement through protein engineering methods, with a view to the creation of an efficient bioinsecticide complex for the specific control of WCR and CPB.

## 5. Materials and Methods

### 5.1. Materials

The aegerolysins (i.e., OlyA6, EryA, PlyA2) and Δ48PlyB (indicated here as PlyB) recombinant proteins were produced as described previously [1,4]. Aliquots of 1 mg/mL aegerolysin were stored in 20 mM Tris-HCl pH 7.5, and aliquots of 0.6 mg/mL PlyB were stored in 20 mM Tris-HCl, 140 mM NaCl, 2% glycerol pH 8.0. The membrane permeabilizing potential of the isolated OlyA6/PlyB, PlyA2/PlyB, and EryA/PlyB was complexes assessed on calcein-loaded artificial lipid vesicles containing 5 mol% of the aegerolysin membrane receptor, the CPE [1]. The protein batches used in this study had a comparable membrane-permeabilizing potential as those used in the toxicity tests with CPB and WCR [1].

### 5.2. Animals

The woodlice *Porcellio scaber* (Crustacea, Isopoda) were collected from a compost heap in a non-contaminated, pollution-free garden in Kamnik, Slovenia (46°13′32.988″ N; 14°36′42.12″ E). Before the experiment, they were maintained for several months under constant temperature (20 ± 2 °C) and illumination (16:8 h light:dark) conditions in a climate-controlled chamber at the University of Ljubljana. The isopods were maintained in glass containers with a mixture of loamy sand and peat at the bottom, and fed with dry leaves of common hazel (*Corylus avellana*), common alder (*Alnus glutinosa*) and carrots. Healthy adult woodlice (30–60 mg fresh body mass) of both sexes were used in the experiments. Molting individuals, and females with marsupia were excluded from the experiments.

The carnolian honeybees *Apis mellifera carnica*, Pollman 1879 (Insecta, Hymenoptera: Apidae) were collected in June and July 2020 from healthy colonies at the Department of Biology, Biotechnical Faculty, University of Ljubljana. They were maintained according to good honeybee practice, and were not treated with any chemical substance for 1 month prior to the experiments. For each experiment, adult worker honeybee foragers were collected from the entrance of the hives using an aspirator, at approximately the same hour, between 10 and 12 a.m. The honeybees were randomly distributed into test cages made of wood and steel wire mesh, and with a sliding transparent glass opening, as described in detail by Glavan et al. [42]. During the collection, the bees already transferred to the cages were immediately provided *ad libitum* with dechlorinated water and 1.5 M sucrose solution. On average, 10–15 honeybees were collected per cage, and the cages with the bees were transferred to an incubator (30 °C, 60% relative humidity) and provided ad libitum dechlorinated water for 2 h.

Twenty 9-week-old male Balb/c mice (*Mus musculus*, Rodentia, Mammalia) weighing 24.4 ± 0.90 g were originally obtained from Envigo RMS Srl (Udine, Italy) and bred at the Veterinary Faculty, University of Ljubljana, under standard conditions, and with a 12–12 h light/dark cycle at 22 to 24 °C and 40 to 60% humidity. They were fed with a standard maintenance chow with a fixed formula (Envigo RMS Srl, Udine, Italy), and had potable tap water available ad libitum.

### 5.3. Toxicity Testing

#### 5.3.1. Woodlice (*Porcellio scaber*)

The woodlice were exposed to the test substances over a 7-day feeding experiment with potato (*Solanum tuberosum*) leaves. The leaves were collected from adult plants that had not been sprayed with any pesticide. Fresh leaves were cut into 14-mm-diameter disks and immersed for 5 min into a solution of each of the aegerolysins (0.5 mg/mL) with PlyB (0.04 mg/mL). This defined a concentration of 9.0 µg/cm^2^ aegerolysin/PlyB on each leaf surface. The protein concentrations were chosen based on our previous studies of the effects of *Pleurotus* aegerolysin/PlyB complexes on WCR and CPB [1]. All of the protein mixtures were prepared in 20 mM Tris-HCl, 0.5% glycerol, pH 7.5. The aegerolysins or PlyB alone were not tested with the woodlice. The OlyA6/PlyB, PlyA2/PlyB and EryA/PlyB complexes in in 20 mM Tris-HCl, 0.5% glycerol, pH 7.5 were also incubated apart at room temperature, and their activity was monitored before and after the experiments using a hemolysis assay with bovine erythrocytes [4,43], or using a calcein release test from small unilamellar lipid vesicles containing 5 mol% CPE [1]. The protein mixtures did not lose their membrane permeabilizing potential during the 7-days period of the toxicity tests.

The dry leaf disks were weighed before the application of the test substances, with the preparation of 10 replicates per treatment (plus the control). The control group received leaf discs that had been soaked in 20 mM Tris-HCl, 0.5% glycerol pH 7.5. One woodlouse was placed in each plastic Petri dish, and one leaf disk was added to each as food. Water droplets were applied onto the cover of the Petri dish to maintain the humidity. The Petri dishes were incubated in a glass container with constant air humidity (60%), temperature (20 ± 2 °C) and illumination (16:8 h light:dark).

During the experiments, the survival of the woodlice was followed and the moisture of their atmosphere was controlled. After 7 days of exposure, the leaves were air-dried, the feces were removed from the leaves using a brush, and the mass of the leaves consumed per animal fresh mass was calculated. The mass of the air-dried feces was also recorded. The data were analyzed using the OriginPro software (OriginPro 2020, OriginLab Corporation, Northampton, MA, USA). The feeding rates were calculated as the mass of the leaves eaten per animal mass, and the defecation rates as the mass of feces per animal mass. Kruskal-Wallis non-parametric tests were carried out, followed by pairwise comparisons with Mann–Whitney U-tests. Differences with *p* ≤ 0.05 were considered as significant.

#### 5.3.2. Honeybees (*Apis mellifera carnica*)

Following the OECD TG 245 protocol [44], the single aegerolysins and the OlyA6/PlyB, PlyA2/PlyB and EryA/PlyB mixtures were offered to the honeybees ad libitum by dissolving them in 1.5 M sucrose solutions. For this kind of oral exposure, the gravity feeders were graduated 5 mL sterile single-use syringes with cut open ends (polypropylene + polyethylene; Ecoject, Dispomed, Gelnhausen, Germany). These were inserted vertically and extended to the bottom of the cage. Each group of bees was also provided with dechlorinated water during the experiments. The feeders for dechlorinated water were graduated 1 mL sterile single-use syringes with cut open ends, inserted vertically from the tops of the cages.

All of the feeders and their contents were renewed with freshly prepared food every 24 h during the exposure period, to ensure that the honeybees had enough food. The experiments were held in a controlled-climate chamber in continuous darkness at a temperature of 30 ± 0.5 °C, and 60% relative humidity. The OlyA6/PlyB, PlyA2/PlyB and EryA/PlyB complexes in 1.5 M sucrose solutions were also incubated apart at room temperature, and their activity was monitored before and after the experiments using a hemolysis assay with bovine erythrocytes [4,43], or using a calcein release test from small unilamellar lipid vesicles containing 5 mol% CPE [1]. The protein mixtures did not lose their membrane permeabilizing potential during the duration of the toxicity tests. The dead honeybees were counted and removed, and the amounts of consumed solutions were evaluated by weighing the gravity feeders every 24 h.

The first set of tests were performed to compare the effects of honeybee oral exposure to single aegerolysins to the effects of the aegerolysin/PlyB mixtures. The protein concentrations were chosen based on our previous studies of the effects of the *Pleurotus* aegerolysin/PlyB complexes on WCR and CPB [1]. This set of tests consisted of two separate experiments. In the first, the honeybees were exposed to 0.5 mg/mL of the individual aegerolysins (OlyA6, PlyA2, EryA). In the second experiment, the honeybees were exposed to the 0.5/ 0.04 mg/mL OlyA6/PlyB, PlyA2/PlyB or EryA/PlyB mixtures. Each experiment included also a control group of honeybees that were orally exposed to buffer (20 mM Tris, 0.5% glycerol, pH 7.5) mixed with 1.5 M sucrose (1/1, *v/v*). Each experimental group and control group included three cages with honeybees (*n* = 15 per cage; 12 cages used for each of the two experiments). The survival and feeding rates of the honeybees in each cage were checked at 24 h and 48 h of exposure.

The subsequent dose–response analysis was carried out to evaluate the oral 48 h mean lethal concentrations (LC_50_) for the aegerolysins/PlyB mixtures that induced significant mortality of honeybees in the first set of tests. Two dose-response experiments were performed, separately for the OlyA6/PlyB and PlyA2/PlyB complexes. The same concentrations of OlyA6 or PlyA2 in 1.5 M sucrose solution were used for both of these experiments, as: 0, 0.12, 0.172, 0.245, 0.35 and 0.5 mg/mL, with the respective concentrations of PlyB of 0, 0.009, 0.0137, 0.019, 0.028 and 0.04 mg/mL. For each treatment concentration, three cages with honeybees were used (*n* = 10 per cage; 18 cages used for each dose response analysis). The survival and feeding rates were again checked at 24 h and 48 h.

The data were analyzed using Microsoft Excel (2010) and the OriginPro software (OriginPro 2020, OriginLab Corporation, Northampton, MA, USA). The experiments were considered valid if the mortality in the controls did not exceed 15% at the end of the test, as suggested by OECD TG 245. The feeding rates were calculated as the amount of food eaten per number of live honeybees in the cage. Forty-eight hour LC_50_ values were calculated using sigmoidal function according to Logistic Equation (OriginPro 2020, OriginLab Corporation, Northampton, MA, USA) (Figure S1).

#### 5.3.3. Mice (*Mus musculus*)

For the acute toxicity determination in mice, a stock solution of 1 mg/mL EryA and PlyB was prepared at the molar ratio of 40:1, and two additional dilutions were prepared in 20 mM Tris-HCl, 140 mM NaCl and 2% glycerol.

The mice were randomly divided into four experimental groups at 5 mice/group. The compounds were administered intravenously through the right tail vein with 100 µL of the prepared protein mixture, in single doses of 0.5, 1.0 or 3.0 mg/kg body mass. The concentrations were chosen based on our previous experiments of the effects of OlyA6/PlyB isolates on mice [25]. The mice in the control group received an equal volume of the buffer mixture. After injection, the mice were continuously observed for 24 h for signs of toxicity and lethality.

Thereafter, the mice were sacrificed by cervical dislocation and immediate exsanguination. Body and organ weights (liver, lung, right kidney, heart) were recorded to the nearest 0.01 g, and the relative organ mass (g/100 g body mass) calculated.

The EryA/PlyB complexes in 20 mM Tris-HCl, 140 mM NaCl and 2% glycerol were also incubated apart at room temperature, and their activity was monitored before and after the experiments using a calcein release test from small unilamellar lipid vesicles containing 5 mol% CPE [1]. The protein mixtures did not lose their membrane permeabilizing potential during the 24-h duration of the toxicity tests.

Significance was defined as *p* < 0.05. Sigma Plot 12.5 (Systat Software, San Jose, CA, USA) was used for the data analyses. The data are presented as means ± standard deviation. The differences between the groups were analyzed using one-way ANOVA, followed by Bonferroni’s post-hoc tests (for normal distribution of the data).

#### 5.3.4. Histological Analysis of the Mice Organs

The liver, lung, kidney and heart samples were first fixed in 10% buffered formalin (Shandon Formal-Fixx 10% neutral buffered formalin; Thermo Scientific) and then dehydrated and embedded in paraffin using the standard procedure. Subsequently, 5 µm histological sections were cut and stained with hematoxylin and eosin. Histology evaluation was performed using a light microscope (Ni/U; Nikon) equipped with a digital camera (DS-Fi1) and the NIS-Elements BR 4.60 imaging software (Nikon Instruments Europe B.V., Badhoevedorp, The Netherlands). Representative images are presented using Adobe Creative Cloud (Adobe Inc., San Jose, CA, USA).

## Figures and Tables

**Table 1:** Body and organ mass of mice after 24 h exposure to increasing doses of intravenous EryA/PlyB (40:1 molar ratio).

EryA/PlyB	Body Mass	Relative Organ Mass (% Body Mass)			
(mg/kg)	(g)	Liver	Lung	Kidney	Heart
0	23.56 ± 0.41	4.33 ± 0.16	0.82 ± 0.25	0.67 ± 0.06	0.58 ± 0.05
0.5	24.40 ± 0.52	4.07 ± 0.05	0.83 ± 0.24	0.71 ± 0.02	0.57 ± 0.07
1.0	24.46 ± 1.53	4.25 ± 0.23	0.87 ± 0.14	0.67 ± 0.03	0.63 ± 0.13
3.0	24.50 ± 0.54	4.15 ± 0.15	0.83 ± 0.17	0.69 ± 0.02	0.58 ± 0.05
*p* value	0.304	0.097	0.977	0.245	0.720

Data are means ± standard deviation (*n* = 5 mice per group).

## Data Availability

All of the data generated or analyzed during this study are included in this published article.

## Supplementary Materials

The following are available online at https://www.mdpi.com/article/10.3390/toxins13070457/s1, Figure S1. Data for survival rates of honeybees feeding on increasing concentrations of OlyA6/PlyB and PlyA2/PlyB for 48 h, and sigmoidal function fit curves used to derive LC50 data.
