# Supplementary material for: Effects of Bioinsecticidal Aegerolysin-Based Cytolytic Complexes on Non-Target Organisms
Source: Toxins (Basel). 2021 Jun 30;13(7):457. doi: 10.3390/toxins13070457 (PMC8310125; doi:10.3390/toxins13070457)
Supplement: Supplementary file 1 [file toxins-13-00457-s001.zip › toxins-1265189-supplementary.pdf]

toxins

Supplementary Materials: Effects of Bioinsecticidal Aegerolysin-Based Cytolytic Complexes on Non-Target Organisms

Anastasija Panevska, Gordana Glavan, Anita Jemec Kokalj, Veronika Kukuljan, Tomaž Trobec, Monika Cecilija Žužek, Milka Vrecl, Damjana Drobne, Robert Frangež and Kristina Sepčić

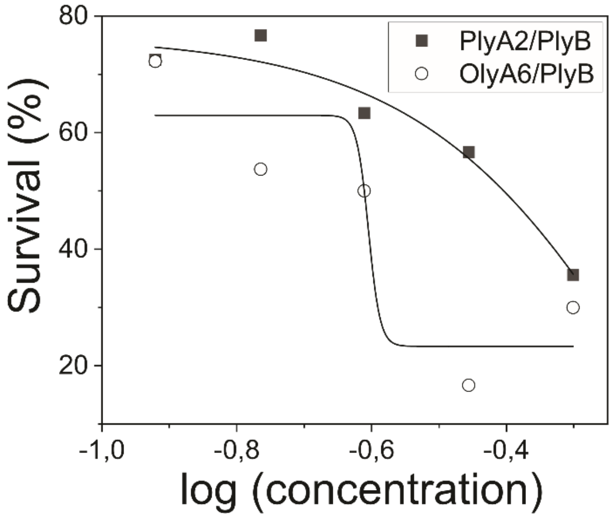

Figure S1. Data for survival rates of honeybees feeding on increasing concentrations of OlyA6/PlyB and PlyA2/PlyB for 48 h, and sigmoidal function fit curves used to derive LC_{50} data.
